# Combining Clinicopathological Parameters and Molecular Indicators to Predict Lymph Node Metastasis in Endometrioid Type Endometrial Adenocarcinoma

**DOI:** 10.3389/fonc.2021.682925

**Published:** 2021-08-04

**Authors:** Peng Jiang, Yuzhen Huang, Yuan Tu, Ning Li, Wei Kong, Feiyao Di, Shan Jiang, Jingni Zhang, Qianlin Yi, Rui Yuan

**Affiliations:** Department of Gynecology, The First Affiliated Hospital of Chongqing Medical University, Chongqing, China

**Keywords:** combined predictors, endometrial cancer, lymph node metastasis, nomogram, predict

## Abstract

**Background:**

Lymph node metastasis (LNM) is a critical unfavorable prognostic factor in endometrial cancer (EC). At present, models involving molecular indicators that accurately predict LNM are still uncommon. We addressed this gap by developing nomograms to individualize the risk of LNM in EC and to identify a low-risk group for LNM.

**Methods:**

In all, 776 patients who underwent comprehensive surgical staging with pelvic lymphadenectomy at the First Affiliated Hospital of Chongqing Medical University were divided into a training cohort (used for building the model) and a validation cohort (used for validating the model) according to a predefined ratio of 7:3. Logistics regression analysis was used in the training cohort to screen out predictors related to LNM, after which a nomogram was developed to predict LNM in patients with EC. A calibration curve and consistency index (C-index) were used to estimate the performance of the model. A receiver operating characteristic (ROC) curve and Youden index were used to determine the optimal threshold of the risk probability of LNM predicted by the model proposed in this study. Then, the prediction performance of different models and their discrimination abilities for identifying low-risk patients were compared.

**Result:**

LNM occurred in 87 and 42 patients in the training and validation cohorts, respectively. Multivariate logistic regression analysis showed that histological grade (P=0.022), myometrial invasion (P=0.002), lymphovascular space invasion (LVSI) (P=0.001), serum CA125 (P=0.008), Ki67 (P=0.012), estrogen receptor (ER) (0.009), and P53 (P=0.003) were associated with LNM; a nomogram was then successfully established on this basis. The internal and external calibration curves showed that the model fits well, and the C-index showed that the prediction accuracy of the model proposed in this study was better than that of the other models (the C-index of the training and validation cohorts was 0.90 and 0.91, respectively). The optimal threshold of the risk probability of LNM predicted by the model was 0.18. Based on this threshold, the model showed good discrimination for identifying low-risk patients.

**Conclusion:**

Combining molecular indicators based on classical clinical parameters can predict LNM of patients with EC more accurately. The nomogram proposed in this study showed good discrimination for identifying low-risk patients with LNM.

## Introduction

Endometrial cancer (EC) is a common gynecological malignant tumor with a high overall survival rate. Specifically, in patients with early low-risk EC, the 5-year overall survival rate is higher than 80% (1). However, some patients still experience relapse (2). Lymph node metastasis (LNM) is a significant risk factor for the prognosis of these patients (3). Recently, the role of systematic lymph node dissection in the treatment of EC has become controversial (4). Random tests have shown that conventional lymph node dissection does not lead to a survival benefit in patients with early-stage EC (3). In contrast, for some patients with early-stage EC, the incidence of complications, including lymphatic cysts, deep vein thrombosis, and intestinal obstruction, increases (5), thus increasing hospitalization expenses and the need for medical resources (6). This has led to the most current international guidelines to no longer recommend systematic lymph node dissection for patients with type I (endometrioid histologic type) EC. They are at low or intermediate risk for recurrence (1). To endorse a balance between over- and under-treatment, many strategies for selecting patients in whom lymph node dissection may be omitted have been investigated and proposed in the past decade, including various risk stratification systems and prediction models for LNM. Currently, most risk stratification systems and prediction models are based on classical clinicopathological parameters (3, 7). For example, the Mayo Risk Stratification model (8) defines patients with low-risk LNM in whom lymph node dissection could be omitted, which would include grade 1 or 2 endometrioid EC, tumor diameter (TD) < 20mm, and myometrial invasion (MI) < 50%. Sofiane et al. (3) established a nomogram involving four clinicopathological parameters (histological grade, lymphovascular space invasion, TD, and MI) to predict LNM in EC. However, it seems that these risk stratification systems and prediction models can no longer accurately predict LNM (9). One study (10) reported that 10% of patients at low risk and 15% of patients at intermediate risk of recurrence had nodal metastases based on the current risk stratification system. This means that even in early type I EC, some patients may not be adequately treated, which may significantly impact postoperative management and adjuvant treatment indications. Therefore, adding predictive indexes with potential prognostic value based on existing models to increase prediction performance and discrimination is crucial (11).

The serological indicator cancer antigen 125 (CA125) is a tumor marker with good sensitivity closely related to the prognosis of endometrial cancer, especially in EC patients with abdominal metastasis (12). Several studies (4, 11) have shown that CA125 can predict LNM in EC. Different cutoff values of CA125 have been determined to better predict LNM. Currently, the clinically recognized and widely used cutoff value of CA125 in EC is 35 U/ml (12, 13). ER, PR, Ki67, and P53 are commonly used immunohistochemical markers in clinical practice (14). Many studies have shown that the loss of ER and PR, the increase in the Ki67 index, and the abnormal expression of P53 protein lead to a poor prognosis of EC (15, 16). Therefore, these markers are usually used as predictors in the evaluation of LNM and EC recurrence (17). Currently, comprehensive prediction models that combine clinicopathological parameters, immunohistochemical markers, and serological indicators are rare. This study established a nomogram that incorporates these three types of parameters to predict LNM in EC and evaluate which EC patients would benefit from lymph node dissection.

## Materials and Methods

### Study Population

Data from hospitalized patients with FIGO stages I to III [2009 guidelines (18)] endometrial cancer who underwent initial surgical treatment at the First Affiliated Hospital of Chongqing Medical University from October 2013 through June 2020 were collected. Age, body mass index, FIGO stage, the preoperative serum indicator CA125, histological type and grade, depth of myometrial invasion, cervical stromal infiltration, LVSI status, number of removed pelvic and para-aortic lymph nodes, presence of LNM, and immunohistochemistry results of four immunohistochemical markers (ER, PR, Ki67, and P53) were collected for each patient. Patients with endometrioid carcinoma, incomplete medical records, administration of preoperative adjuvant therapy, other malignant tumors, no standard surgical treatment or lymph node resection, or lack of regular follow-up after surgery were excluded.

### Treatment and Follow-Up

All patients included in this study received comprehensive surgical staging, including hysterectomy with bilateral salpingo-oophorectomy + systematic pelvic lymphadenectomy ± sentinel lymph node (SLN) biopsy ± para-aortic lymphadenectomy. Systematic pelvic and para-aortic lymph node dissection was recommended for patients with high-risk factors, including grade 3 type 1 EC, deep myometrial invasion, and pelvic sentinel lymph node metastasis seen on intraoperative histological examination or final histological examination (3). According to the criteria proposed by AlHilli et al., removal of at least 10 pelvic LNs with or without five para-aortic LNs was defined as effective lymph node dissection (13). The need for adjuvant treatment (supplementary radiotherapy or even combined chemotherapy) was determined by international guidelines (19) and multidisciplinary discussion after surgery. Follow-up was performed every 3 months for the first 2 years after surgery, every 6 months for the next 3 years, and once a year thereafter. The follow-up plan included regular physical examinations and necessary auxiliary examinations, including routine biochemical tests, imaging examinations, and histological examinations. Recurrence was defined as vaginal stump recurrence, central pelvic region recurrence, peritoneal metastasis, and distant metastasis (20), confirmed by tissue diagnosis whenever possible (21). Recurrence-free survival (RFS) was defined as the time between the complete removal of the malignant tumor and the date of recurrence (confirmed by histology or radiology). Overall survival (OS) was defined as the time from primary surgery to death from any cause (2).

### Postoperative Pathology and Immunohistochemistry

All postoperative specimens were processed according to the same standards (22) at the Pathology Laboratory Center of Chongqing Medical University. The specimens were fixed in formalin and embedded in paraffin. H&E staining was used to confirm the cancerous areas. The histological type of tumors, histological grade of endometrioid (type I) endometrial carcinoma, the size of the lesion, and the range of infiltration were initially judged by a primary pathologist at the center and reviewed by an expert physician. Immunohistochemistry was performed in an automatic immunostaining machine (Leica Bond-Max, Milton Keynes, UK). ER (Clone 1D5, 1:50), PR (Clone PgR636, 1:500), Ki67 (Clone MIB1, 1:300), and P53 (Clone DO7, 1:200) antibodies were used for immunohistochemistry. The results of ER, PR, Ki67, and P53 immunostaining were initially independently assessed by two experienced pathologists and recorded as a percentage of positively stained tumor cells (0–100%). The assessments of the proportion of positive tumor cells by pathologists were considered consistent if the difference did not exceed 10%. If the initial assessment of the proportion varied by more than 10%, the results were reassessed (unblinded) to reach a consensus. Finally, the two proportions assessed by the two pathologists were averaged to represent the final result of the proportion of positive tumor cells (16).

The ER, PR, and Ki67 results were considered continuous variables (percentage of positively stained tumor cells, 0–100%) rather than binary variables (positive or negative) in this study, which also fit the proposed model (nomogram). For the results of P53 immunohistochemistry, as suggested by the three-tier system for P53 immunohistochemistry interpretation (23), overexpression (proportion of positive tumor cells ≥75%), and complete deletion (proportion of positive tumor cells 0%) were both considered abnormal (mutation-type) expression, whereas normal (wild-type) P53 expression levels were defined to be between these two levels (0–75%).

### Statistical Analysis

Patients enrolled in this study were randomly divided into a training cohort and a validation cohort by R software according to a predefined ratio of 7:3, consistent with many other similar studies (2, 24, 25). The training cohort was used for construction and internal verification of the model. In contrast, the validation cohort was used for external verification of the model. The balance and consistency of the data distribution between the two cohorts were compared. Categorical variables were expressed as frequencies and percentages, and the chi-square test was used for comparisons between groups. Continuous variables are represented by the mean, median, and range, and comparisons between groups were performed by Student’s t-test and rank-sum test. P < 0.05 was considered statistically significant.

In the training cohort, univariate logistic regression analysis was used to analyze the correlation between each prognostic factor and LNM, and factors for which P < 0.05 in the univariate analysis were further included in the multivariate logistic regression analysis. Finally, factors for which P < 0.05 in the multivariate analysis were used to develop the model using R software.

Internal and external validations of the model were performed in the training and validation cohorts, respectively. A calibration curve was used to evaluate the fitness of the model, and the consistency index (C-index) was further used to compare the prediction accuracy between different models. The C-index is mainly used to evaluate the predictive performance of the model (26), which ranges from 0 to 1; the model is considered to have poor, fair, or good performance if the C-index lies between 0.5 and 0.6, 0.6, and 0.7 or is greater than 0.8, respectively (3).

The optimal threshold of probability of LNM predicted by the model was determined by the receiver operating characteristic (ROC) curve and Youden index (Youden index = sensitivity + specificity −1) (27). Patients were divided into a high-risk group and a low-risk group for LNM according to the threshold. Similar to other studies (11), the proportion of the low-risk population, sensitivity, specificity, positive predictive value (PPV), and negative predictive value (NPV) were calculated to compare the ability to distinguish high-risk and low-risk patients using different models. Sensitivity was defined as the proportion of patients with LNM correctly identified by the model among the patients who had LNM. Specificity was defined as the proportion of patients without LNM correctly identified by the model among the patients who had no LNM. PPV was calculated as the proportion of patients with LNM in the high-risk group of LNM determined by the model. NPV was calculated as the proportion of patients without LNM in the low-risk group identified by the model. Finally, Kaplan-Meier analysis and the log-rank test were used to describe the distribution of RFS and OS in the high-risk and low-risk groups. The data were analyzed using SPSS software (version 25.0, IBM Statistics, Chicago, IL, USA) and R software (version 4.0.3, http://www.r-project.org) (**Supplementary Material**).

## Results

### Patient Characteristics

**Figure 1:**
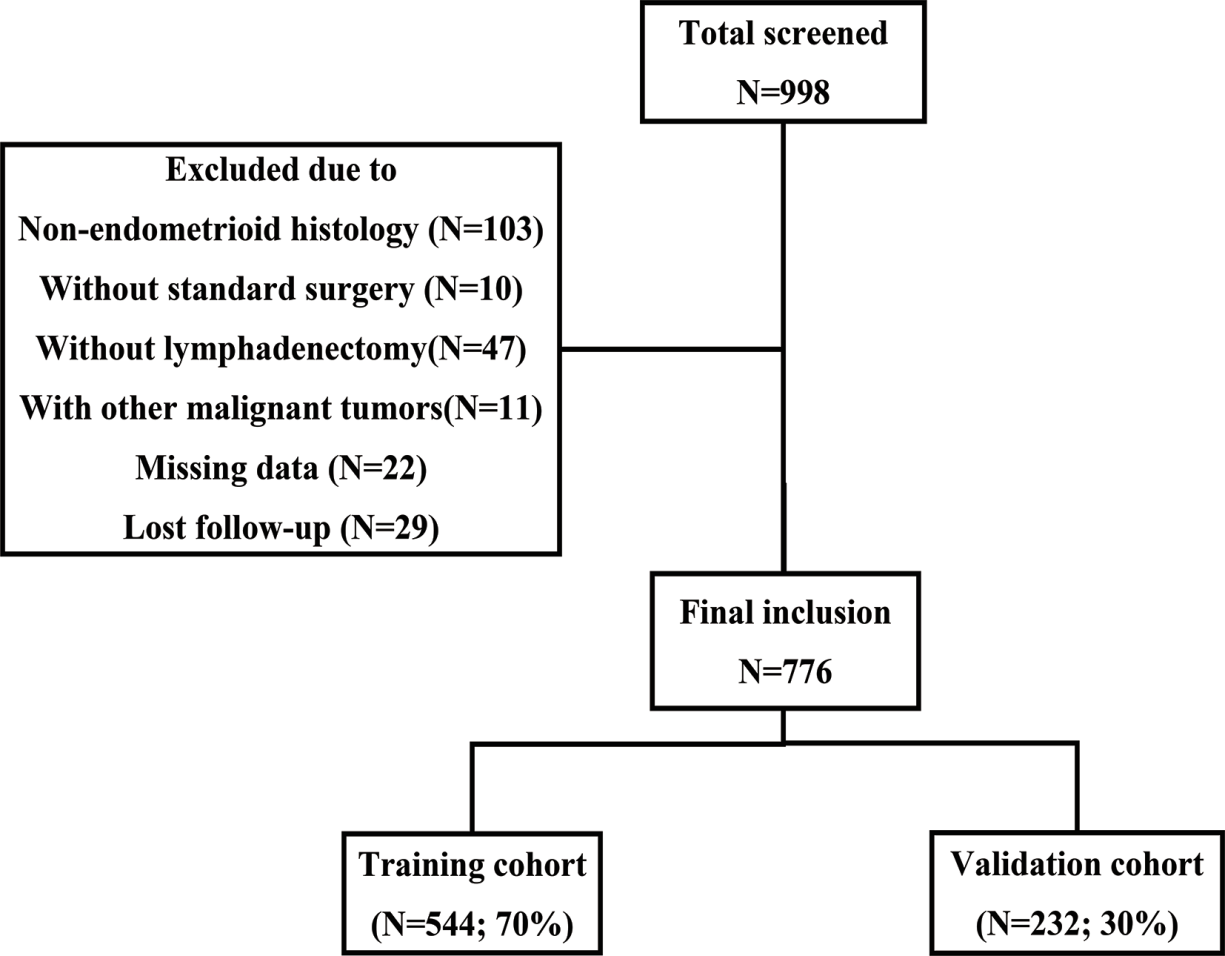
Flowchart of patient inclusion.

A total of 998 patients with stage I-III EC received initial surgical treatment at the First Affiliated Hospital of Chongqing Medical University between October 2013 and June 2020. Among these patients, 10 retained their ovaries because of early-stage disease and fertility requirements, and another 47 did not undergo lymph node dissection. Finally, 776 patients were included according to the inclusion and exclusion criteria (**Figure 1**). **Table 1** summarizes the baseline characteristics of the training and validation cohorts. No statistically significant difference was found in the baseline characteristics between the two cohorts, which ensured that it would not be affected by confounding factors between the two cohorts when externally verifying the model in the validation cohort. Most patients had stage I disease (82.2% of the training cohort and 84.5% of the validation cohort). In the training cohort, 163 (30.0%) patients underwent abdominal para-aortic lymph node dissection, and a total of 87 (16.0%) patients had LNM, among which 15 had abdominal para-aortic LNM. In the validation group, 61 (26.3%) patients underwent abdominal para-aortic lymph node dissection, and 42 (18.1%) patients had LNM, and of these, six had abdominal para-aortic LNM. No patient in the two cohorts had isolated para-aortic LNM.

**Table 1:** Baseline characteristics of the training and validation cohorts.

Variable	Training cohort, N = 544	%	Validation cohort, N = 232	%	P value
**Age (yrs)**					0.557
Mean (± SD)	53.77 (± 9.28)		53.34 (± 9.20)		
Median (range)	53.00(25-81)		52.00 (24-81)		
**BMI (kg/m^2^)**					0.942
Mean (± SD)	24.64 (± 3.72)		24.66 (± 3.71)		
Median (range)	24.24 (16.53–41.87)		24.45 (16.35–45.72)		
**Histologic grade**					0.463
1	136	25.0	64	27.6	
2	275	50.6	106	45.7	
3	133	24.4	62	26.7	
**Myometrial invasion**					0.832
<1/2	377	69.3	159	68.5	
≥1/2	167	30.7	73	31.5	
**Cervical stromal invasion**					0.434
No	447	82.2	196	84.5	
Yes	97	17.8	36	15.5	
**LVSI**					0.493
LVSI-negative	412	75.7	181	78.0	
LVSI-positive	132	24.3	51	22.0	
**Serum CA125 (U/ml)**					0.772
<35	411	75.6	173	74.6	
≥35	133	24.4	59	25.4	
**Ki67 positivity ratio (%)**					0.201
Mean (± SD)	34.76 (± 20.07)		32.86 (± 19.39)		
Median (range)	30.00 (0–90)		30.00 (1–80)		
**ER positivity ratio (%)**					0.716
Mean (± SD)	62.40 (± 35.48)		62.46 (± 34.99)		
Median (range)	90.00 (0–95)		82.50 (0–90)		
**PR positivity ratio (%)**					0.740
Mean (± SD)	61.31 (± 36.11)		61.13 (± 36.72)		
Median (range)	80.00 (0–95)		80.00 (0–95)		
**P53 expression**					0.821
Normal	340	62.5	143	61.6	
Abnormal	204	37.5	89	38.4	
**Scope of lymphadenectomy**					0.302
Only pelvic LNs	381	70.0	171	73.7	
Pelvic + para-aortic LNs	163	30.0	61	26.3	
**Number of LNs removed**					0.645
Mean (± SD)	33.76 (± 14.85)		33.23 (± 14.43)		
Median (range)	32.00 (10–119)		32.00 (10–91)		
**LN metastasis**	87	16.0	42	18.1	0.470
Only pelvic LN metastasis	72		36		
Pelvic + para-aortic LN metastasis	15		6		

BMI, body mass index; LVSI, lymphovascular space invasion; ER, estrogen receptor; PR, progesterone receptor; LN, lymph node.

### Univariate and Multivariate Logistic Regression Analyses of Predictive Factors for LNM

Univariate and multivariate logistic regression analyses were performed to screen candidate predictors (**Table 2**). In the univariate logistic regression analysis, histological grade, myometrial invasion, cervical stromal invasion, LVSI, serum CA125, and all four immunohistochemical markers (Ki67, ER, PR, and P53) were associated with LNM. However, the multivariate logistic regression analysis did not find that cervical stromal invasion and PR were associated with LNM, and thus, these two candidates were excluded from the model. The remaining predictors, including histological grade (P = 0.022), myometrial invasion (P = 0.002), LVSI (P = 0.001), serum CA125 (P = 0.008), Ki67 (P = 0.012), ER (P = 0.009), and P53 (P = 0.003) still demonstrated statistically significant correlations with LNM, and thus, these seven predictors were further used for constructing predictive models.

**Table 2:** Univariate and multivariate analyses of predictive factors for lymph node metastases in the training cohort.

Variables	Univariate analysis			Multivariate analysis		
	Hazard ratio	95% CI	P value	Hazard ratio	95% CI	P value
Histologic grade						
1	1.000		<0.001	1.000		0.072
2	5.130	1.789–14.709	0.002	2.892	0.967-8.650	0.058
3	17.448	6.064–50.209	<0.001	3.938	1.219-12.720	0.022
Myometrial invasion (≥1/2 *vs* <1/2)	4.165	2.588–6.702	<0.001	2.457	1.401-4.310	0.002
Cervical stromal invasion (Yes *vs* No)	3.509	2.110–5.836	<0.001	1.293	0.682–2.453	0.431
LVSI (Positive *vs* Negative)	4.840	2.989–7.837	<0.001	2.625	1.460–4.720	0.001
CA125 (≥35 *vs* <35)	3.331	2.063–5.379	<0.001	2.213	1.227–3.994	0.008
Ki67 positivity ratio (%)	1.031	1.020–1.043	<0.001	1.017	1.004–1.031	0.012
ER positivity ratio (%)	0.975	0.969–0.981	<0.001	0.987	0.977–0.997	0.009
PR positivity ratio (%)	0.979	0.973–0.985	<0.001	0.997	0.987–1.006	0.503
P53 expression (abnormal *vs* normal)	1.899	1.196–3.015	0.007	2.407	1.346–4.303	0.003

LVSI, lymphovascular space invasion; ER, estrogen receptor; PR, progesterone receptor.

### Establishment of the Model and Evaluation of Its Performance

**Figure 2:**
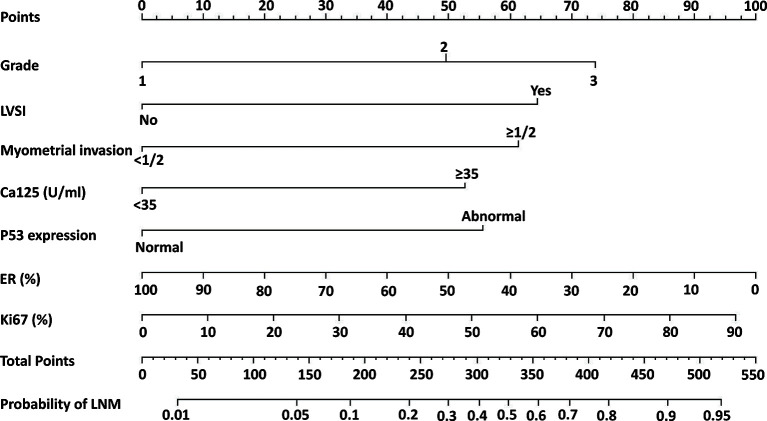
Nomogram model for estimating the probability of LNM in women with endometrial cancer. To estimate the probability of LNM, locate the patient’s grade on the “grade” axis. Draw a straight line up to the “point” axis to determine the points for grade. Repeat the process for each of the remaining axes, drawing a straight line each time to the “point” axis. Add the points received from each variable and locate this number on the “total point” axis. A straight line is drawn down from the “total point” axis to the “probability of LNM” axis to determine the risk of LNM in patients.

**Figure 3:**
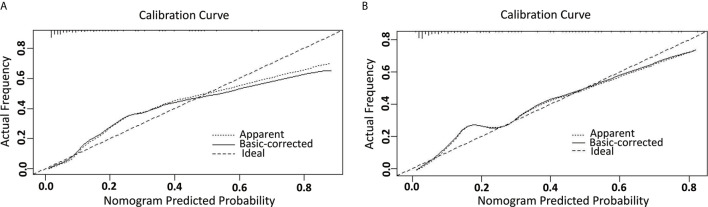
The calibration curve for internal and external validation of the nomogram model. **(A)** The internal calibration curve for the nomogram of predicting LNM in EC; **(B)** The external calibration curve for the nomogram of predicting LNM in EC.

A nomogram model was established to assess the probability of LNM in EC (**Figure 2**). The length of the line segment of each predictor in the nomogram represents the weight of causing the resulting event (LNM). As shown in the figure, the predictive value (the weight) of immunohistochemical markers was still very considerable compared with the classical clinicopathological parameters, especially ER and Ki67. The calibration curve of the model also showed good fitness in both internal and external verifications (**Figure 3**). Finally, the C-index of several different models was calculated to compare their accuracy in predicting LNM (**Table 3**). As shown in the table, each model was composed of various prognostic indicators, including classical clinicopathological parameters, serological indicator, or immunohistochemical markers. The models that included classical clinicopathological parameters (Model A (3), Model B (28), Model E (29), and the model proposed in this study) had good discrimination (C-index of these models was ≥ 0.80). In contrast, the models that did not include classical clinicopathological parameters [Model C (15) and Model D (11)] had relatively poor discrimination (C-index of the two models was < 0.80), indicating that classical clinicopathological parameters were still the leading reference indicators for predicting LNM in EC. Moreover, the model proposed in this study had better discrimination than other models. The C-index for internal verification and external verification was 0.90 (95% CI, 0.87–0.94) and 0.91 (95% CI, 0.86–0.96), which also implied that adding specific markers with prognostic value, such as the serological indicator CA125 and immunohistochemical markers, to the classical clinicopathological parameters can improve the predictive performance of the model.

**Table 3:** The discriminatory power (C-index) of different models in the training and validation cohorts.

Model	Author	Combination	Training cohort	Validation cohort
			C-index (95%CI)	C-index (95%CI)
Model A (3)	Sofiane Bendifallah et al.	Only classical clinicopathological parameters: pathological grade, LVSI, myometrial invasion, et al.	0.80 (0.75–0.85)	0.86 (0.81–0.91)
Model B (28)	Jisun Lee et al.	Classical clinicopathological parameters + serological markers: pathological grade, myometrial invasion + serum CA125	0.80 (0.75–0.84)	0.84 (0.77–0.90)
Model C (15)	Varol Gülseren et al.	Only immunohistochemical markers: Ki67, ER, PR, P53	0.79 (0.74–0.84)	0.73 (0.64–0.81)
Model D (11)	Bingyi Yang et al.	Immunohistochemical markers + serological markers: PR, Ki67 + serum CA125	0.77 (0.73–0.82)	0.71 (0.62–0.79)
Model E (29)	Marcos Ballester et al.	Classical clinicopathological parameters + Immunohistochemical markers: pathological type and grade, LVSI, myometrial invasion + ER, PR	0.85 (0.81–0.89)	0.87 (0.82–0.93)
Model proposed in this study		Classic clinicopathological parameters + serological markers + immunohistochemical markers: histological grade, LVSI, myometrial invasion + serum CA125 + Ki67, ER, P53	0.90 (0.87–0.94)	0.91 (0.86–0.96)

LVSI, lymphovascular space invasion; ER, estrogen receptor; PR, progesterone receptor.

### Optimal Threshold of the Model

**Figure 4:**
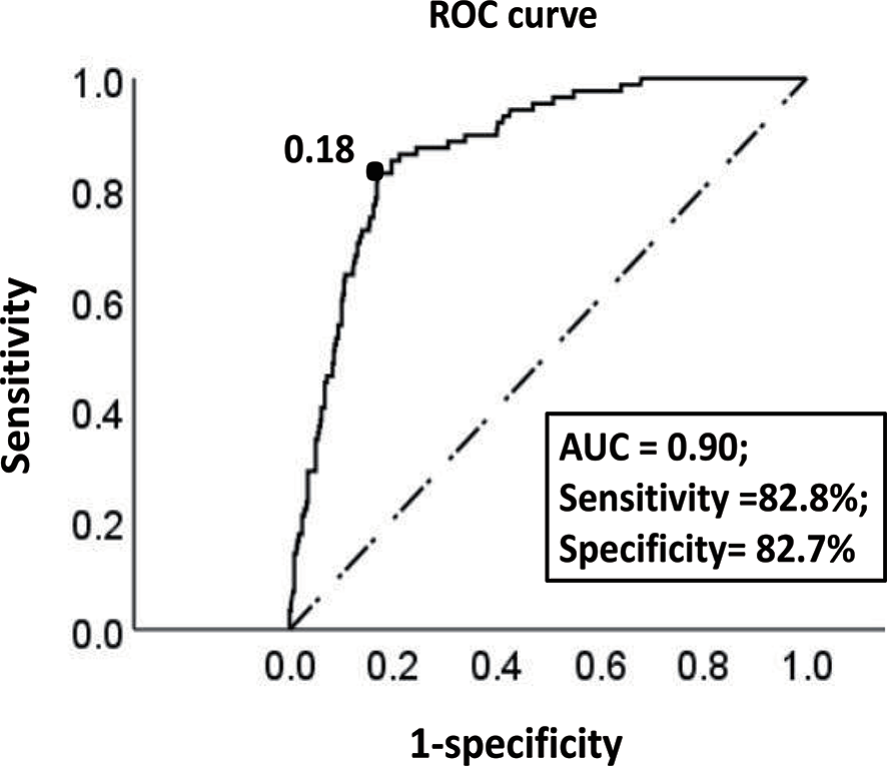
The ROC curve of the optimal threshold value of the probability of LNM predicted by the model. The area under the curve at the “black dot” is the largest, which suggests that the optimal threshold value of the probability of LNM predicted by the model is 0.18 (area under the curve = 0.90; sensitivity, 82.8%; specificity, 82.7%) (dotted line: reference line; solid line: the ROC curve of the model).

In the training cohort, the ROC curve and Youden index indicated that the optimal threshold of the probability of LNM predicted by the model was 0.18 (area under the curve = 0.90; sensitivity = 82.8%; specificity = 82.7%) (**Figure 4**). The risk probability of LNM for all patients was calculated. Patients with a risk probability ≥ 0.18 and < 0.18 were defined as the high-risk and low-risk LNM groups, respectively. According to the threshold, in the training cohort and validation cohort, 72.2% (393/544) (sensitivity = 82.8%, specificity = 82.7%, PPV = 47.7%, NPV = 96.2%) and 67.2% (152/232) (sensitivity = 95.2%, specificity = 80.2%, PPV = 52.6%, NPV = 98.7%) of patients, respectively, were classified as the low-risk group for LNM. For models that predict the risk probability of LNM, in addition to the accuracy of the model’s prediction, the ability to identify the largest group of patients with a low risk of LNM was also crucial. The ability of different models to identify patients in the low-risk group was compared (**Table 4**). By comprehensively comparing various indicators (proportion of low-risk group, sensitivity, specificity, PPV, NPV), our model was significantly better than or close to other models, indicating that the ability of our model to identify the low-risk group of patients was determined to be satisfactory.

**Table 4:** Discrimination of different models in their ability to distinguish patients with a low risk of LNM.

Model	Criteria for low risk of LNM	Proportion of low-risk group	Number of LNM in low-risk group	Sensitivity	Specificity	PPV	NPV
Model A (3)	Probability of LNM calculated by the nomogram <0.2	73.4% (384/523)	18 (65 in total)	72.3% *	79.9%	33.8% *	95.3%
Model B (28)	Pathological grade 1; Myometrial invasion <1/2; Serum CA125 <35 IU/ml	51.7% (89/172) *	1 (18 in total)	94.4% *	57.1% *	20.5% *	98.9%
Model C (15)	Ratio of [(P53 + Ki67)/(ER + PR)] < 0.71	78.1% (375/480)	28 (57 in total)	50.9% *	82.0%	27.6% *	92.5% *
Model D (11)	Serum CA125 < 30.0 IU/mL, PR > 50% and Ki67 < 40%.	61.9% (229/370) *	6 (39 in total)	84.6% *	67.4% *	23.4% *	97.3%
Model E (29)	Endometrioid histology; For FIGO stage IA grade 1 or 2: 1) ER ≥30%; 2) ER < 30% and PR ≥15%. For FIGO stage IA grade 3, or FIGO stage IB grade 1 or 2: 1) no LVSI; 2) LVSI and PR ≥15%;	72.7% (346/476)	15 (58 in total)	74.1% *	79.2%	33.1% *	95.7%
Model proposed in this study	Probability of LNM calculated by the nomogram <0.18	72.2% (393/544) in training cohort	15 (87 in total)	82.8%	82.7%	47.7%	96.2%
		67.2% (156/232) in validation cohort	2 (42 in total)	95.2%	80.2%	52.6%	98.7%

NPV, negative predictive value; LVSI, lymphovascular space invasion; ER, estrogen receptor; PR, progesterone receptor. *P < 0.05 compared with the model proposed in this study.

**Figure 5:**
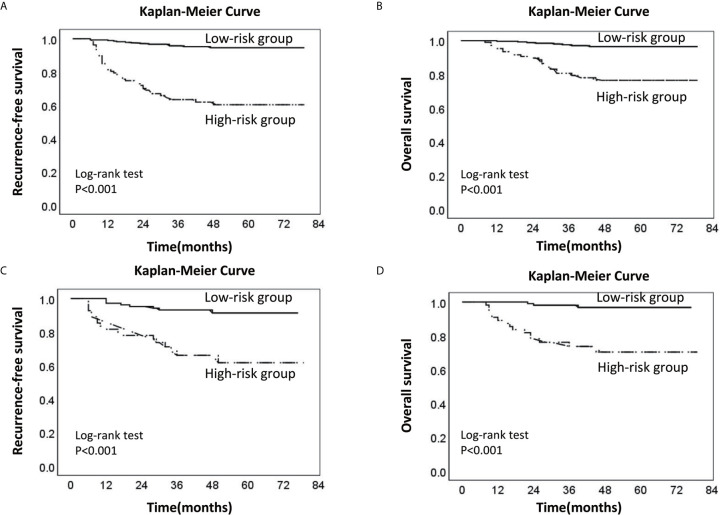
Kaplan-Meier survival curve of the low-risk and high-risk groups of LNM. **(A)** Recurrence-free survival curve of the low-risk and high-risk groups in the training cohort; **(B)** Overall survival curve of the low-risk and high-risk groups in the training cohort. **(C)** Recurrence-free survival curve of the low-risk and high-risk groups in the validation cohort. **(D)** Overall survival curve of the low-risk and high-risk groups in the validation cohort (the dotted line: High-risk group; the solid line: Low-risk group).

Finally, we collected the prognostic information of patients in the two cohorts with a follow-up time of more than 2 years since most patients with recurrence relapsed within 2 years after surgery (**Table 5**). In the training cohort, 53 patients relapsed, of which 30 patients died. Of the 30 patients who died, 26 died due to relapse. In the validation cohort, 26 patients relapsed, 18 patients died, and 17 patients died due to relapse. The median follow-up times of the two groups were 48 and 45 months. The RFS and OS rates of the high-risk and low-risk groups were compared in the two cohorts. In the training cohort, the 3-year RFS rates of patients in the high-risk group and low-risk group were 63.4% (95% CI, 54.0–72.8%) and 95.7% (95% CI, 93.2–98.2%), respectively (P < 0.001), whereas the 3-year OS rates were 80.7% (95% CI, 72.9–88.5%) and 97.6% (95% CI, 95.6–99.6%) (P < 0.001). In the validation cohort, the 3-year RFS rates of patients in the high-risk group and low-risk group were 66.1% (95% CI, 52.8–79.4%) and 93.2% (95% CI, 88.3–98.1%), respectively (P < 0.001), whereas the 3-year OS rates were 73.7% (95% CI, 61.7–85.7%) and 98.1% (95% CI, 95.6–100%), respectively (P < 0.001) (**Figure 5**).

**Table 5:** Recurrence characteristics and follow-up of patients with a follow-up time of more than 2 years.

Variable	Training cohort, N =381	%	Validation cohort, N = 160	%	P value
**Recurrence**					
No	328	86.1	134	83.7	0.482
Yes	53	13.9	26	16.3	
**Sites of relapse**	53		26		
Vaginal stump	2	3.8	2	7.7	0.902
Central pelvic region	15	28.3	7	26.9	
Peritoneal metastases	12	22.6	6	23.1	
Metastasis to other organs	24	45.3	11	42.3	
**Death**					
No	351	92.1	142	88.8	0.208
Death due to recurrence	26	6.8	17	10.6	
Death due to other disease	4	1.1	1	0.6	
**RFS time (months)**					
Median	46.00		43.00		0.330
Mean (± SD)	44.88 (± 19.28)		43.12 (± 18.88)		
Range	6-79		6-79		
**Follow-up (months)**					
Median	48.00		45.00		0.332
Mean (± SD)	47.27 (± 17.55)		45.66 (± 17.77)		
Range	8-79		8-79		

RFS, recurrence-free survival.

## Discussion

The current study established a nomogram model involving classical clinicopathological parameters, serological indicators, and immunohistochemical markers to predict LNM in endometrial cancer. Although these indicators are well known, they are commonly used as clinical prognostic indicators and are easy to obtain. Most importantly, the value of their combination is often overlooked, and good models that combine them organically are lacking. The nomogram allows predictors (such as ER and Ki67) to exist in the model as continuous variables (24). Compared with traditional risk stratification systems, the nomogram can simultaneously evaluate multiple predictors and accurately predict the risk probability of LNM instead of simply summarizing it as “high risk” or “low risk.” For example, in a patient with grade 2 (37 points), LVSI (+) (64 points), CA125 <35 U/ml (0 points), deep myometrial invasion (60 points), normal P53 expression (0 points), 60% ER (+) (38 points), 40% Ki67 (+) (42 points), which corresponds to a total score of approximately 240 points, the risk probability of LNM was about 0.2. Therefore, the nomogram is more personalized in predicting the LNM of patients compared with other models. Moreover, the internal and external calibration curves of the nomogram and the comparison between different models showed that the nomogram proposed in this study had better prediction accuracy and consistency.

Although the overall prognosis of patients with endometrial cancer is excellent, a similar study (11) showed that the overall prognosis between patients with and without LNM is very different. The overall recurrence rate of patients with LNM was 48%, whereas that of patients without LNM was 8%. Similarly, the 5-year disease-free survival rates of patients with and without LNM were 54% and 90%, respectively. For patients with a high risk of LNM, systematic lymph node dissection is usually performed during surgery. In contrast, for low-risk patients, the risk of lymph node dissection outweighs the benefits mentioned in the introduction. Therefore, it is essential to correctly distinguish whether the patient belongs to the high-risk group or the low-risk group for LNM before and after surgery, which can also help doctors decide whether adjuvant therapy or further lymph node dissection should be applied.

At present, the National Comprehensive Cancer Network (NCCN) guidelines (30) and the European Society for Medical Oncology (ESMO) guidelines (1) recommend that systematic lymph node dissection be applied in patients with high-risk tumors, such as those with deeply invasive lesions, high-grade histology, and non-endometrioid histological subtypes (especially clear cell carcinoma and serous carcinoma). However, patients without obvious high-risk factors in the early stage do not typically undergo systematic lymph node dissection, whereas studies have shown that many patients still have LNM. In this study, the high-risk group had more patients with LNM than the low-risk LNM group. The overall RFS and OS rates of the high-risk group were much lower than those of the low-risk group, which indicated that they might be good candidates for systematic lymph node dissection. Unlike other prediction models that only include classical clinicopathological parameters or single-category predictors, if a patient’s clinicopathological parameters indicate a relatively good prognosis while immunohistochemical and serological markers suggest a poor prognosis, the patient might still need to undergo systematic lymph node dissection according to the risk stratification of the model in this study. For example, suppose a patient has CA125 ≥35 U/ml (52 points), abnormal P53 expression (55 points), negative ER expression (100 points), and 90% Ki67 (+) (97 points), which corresponds to a total score of 307 points. In that case, her risk probability of LNM is approximately 0.4. This probability is much greater than 0.18 (the optimal threshold for risk stratification of the model), which indicates that this patient might still have to undergo systematic lymph node dissection. According to the survival curve analysis, to reduce the risk of long-term recurrence, patients in the high-risk group might need adequate postoperative adjuvant treatment (radiotherapy and/or chemotherapy) and closer postoperative follow-up. As shown in **Table 4**, the model proposed in this study had considerable ability to identify the largest group of patients with a low risk of LNM by comprehensively comparing various indicators (proportion of low-risk group, sensitivity, specificity, PPV, NPV), which showed that the model could distinguish most patients with low-risk LNM and avoid unnecessary lymph node dissection.

What is worth mentioning is that, in recent years, four novel subgroups of EC in The Cancer Genome Atlas (TCGA) (31), including the POLE-mutated/ultra-mutated (POLEmt) subgroup (prognosis is the best), the microsatellite-instable/hypermutated (MSI) subgroup (prognosis is relatively poor), the copy-number-low/P53-wild-type (P53wt) subgroup (prognosis is similar to that of the MSI group), and the copy-number-high/P53-mutated (P53 mt) subgroup (prognosis is the worst), have become a “hot spot” for evaluating the prognosis of EC because of its subversion of the traditional pathological classification. For example, studies have confirmed that high-grade endometrioid adenocarcinoma has great heterogeneous, POLE mutations are significantly associated with favorable clinical outcomes in high-grade endometrioid endometrial cancer, which implies that high-grade endometrioid endometrial cancer should be reevaluated by molecular parameters (32). Therefore, adding molecular classification to the predictive model is the trend of future research (4). In this study, the abovementioned P53 molecular classification was incorporated into our model in the form of immunohistochemical markers. It has been reported that the results of P53 immunohistochemistry are extremely consistent with the gene mutation status, and the abnormal staining of P53 immunohistochemistry can almost confirm the presence of TP53 mutations (23). Of course, a small group of tumors harbors more than one molecular classifying feature. For example, about 35% of the POLE mutation subgroup tumors are accompanied by P53 mutations and still have a better prognosis than other genomic subgroups, which is also the reason that POLE mutation analysis is performed in preference to P53 mutation analysis in the ProMisE (Proactive Molecular Risk Classifier for Endometrial Cancer) classifier (33, 34). For the patients with both POLE and P53 mutations, the nomogram proposed in this study may overestimate their prognostic risk. However, tumors with POLE mutations are rare, and only about one third of them have P53 mutations, so a tiny proportion of patients may be affected. Meanwhile, P53 immunohistochemistry is more convenient and cost-effective than POLE mutation analysis and is widely used in clinical practice. Hence, the nomogram proposed in this study is practical and can be a preliminary basis for the molecular classification models in the future. Finally, the current NCCN guidelines (30) and ESMO guidelines (1) point out that sentinel lymph node dissection (SLND) is feasible and can be used as a compromise between no dissection and complete dissection, but it is still experimental. In our study, patients whose risk probability of LNM is around the threshold (0.18) of the model may be good candidates for SLND.

The present study had the following limitations. First, this was a single-center retrospective study, and the effectiveness of the model should be further demonstrated by a multicenter prospective study. Second, we used postoperative pathological specimens for analysis. Many studies have shown that postoperative models (based on the final pathological characteristics) of predicting LNM were better than preoperative models (4, 9). Postoperative pathological specimens can provide reliable data on local tumor staging and histological grading. Specific prognostic markers, such as LVSI, require examination of the completely resected uterus to be thoroughly evaluated (13). However, we still recommend that preoperative endometrial biopsy specimens also be used to predict LNM before surgery (11). The consistency between preoperative biopsy specimens and postoperative pathological specimens of the endometrium should be further prospectively studied. Finally, the model proposed in this study incorporates immunohistochemical markers in the form of continuous variables. Currently, no unified standard for interpreting the immunohistochemistry results has been established, and the “hot spot” assessment method of immunohistochemistry is commonly used as in the current study (22, 35). The immunohistochemistry results were independently assessed by two pathologists (double-blinded) to minimize errors caused by subjective factors, but we still suggest that a universal immunohistochemical interpretation standard be established.

In summary, we established a nomogram combining classical clinicopathological parameters, serological indicators, and immunohistochemical markers to predict LNM in EC. Through this model, the risk of LNM in patients can be more accurately predicted, and the low-risk group of patients with LNM can be well distinguished. Therefore, this model can be a reliable reference for the treatment plan and prognosis management of patients with EC.

## Data Availability Statement

The original contributions presented in the study are included in the article/**Supplementary Material**. Further inquiries can be directed to the corresponding author.

## Ethics Statement

The study was approved by Ethics Committee of Chongqing Medical University (IRB number: 2020-192). The patients/participants provided their written informed consent to participate in this study.

## Author Contributions

RY: conceptualization, methodology, and writing—review and editing. PJ: methodology, data curation, investigation, software, formal analysis, writing—original draft preparation, and writing—review and editing. YH: data curation, investigation, and writing—review and editing. YT, NL, WK, FD, SJ, JZ, and QY: Data curation and supervision. All authors critically reviewed the paper and approved the final version.

## Conflict of Interest

The authors declare that the research was conducted in the absence of any commercial or financial relationships that could be construed as a potential conflict of interest.

## Publisher’s Note

All claims expressed in this article are solely those of the authors and do not necessarily represent those of their affiliated organizations, or those of the publisher, the editors and the reviewers. Any product that may be evaluated in this article, or claim that may be made by its manufacturer, is not guaranteed or endorsed by the publisher.
