# Supplementary material for: Combining Clinicopathological Parameters and Molecular Indicators to Predict Lymph Node Metastasis in Endometrioid Type Endometrial Adenocarcinoma
Source: Front Oncol. 2021 Aug 4;11:682925. doi: 10.3389/fonc.2021.682925 (PMC8372407; doi:10.3389/fonc.2021.682925)
Supplement: Supplementary file 1 [file DataSheet_1.docx]

**APPENDIX**

**Related Computerized Programs for Nomogram With R**

library(rms)

**For dividing data sets into training cohort and vilidation cohort**

library(caret)

library(rms)

train <-createDataPartition(y=alldata $Figo,p=0.70,list=FALSE)

traindata <- alldata[train, ]

testdata <- alldata[-train, ]

**For Nomogram**

library(rms)

dd<-datadist(Ltraindata02)

options(datadist="dd")

f <-lrm(LB~LVSI+Ca12501+myometrialinvasion+histotypes+ER+Ki67+P5301,data = Ltraindata02,x=T,y=T)

nom <-nomogram(f, fun=plogis, fun.at=c(.001,.01, .05, seq(.1,.9, by=.1), .95, .99, .999),lp=F, funlabel="Probability of LNM")

plot(nom)

**For Calibration Curve**

library(rms)

f <-lrm(LB~LVSI+Ca12501+myometrialinvasion+histotypes+ER+Ki67+P5301,data = Ltraindata02,x=T,y=T)

cal <- calibrate(f,method = "boot",B = 1000)

plot(cal, xlab = "Nomogram Predicted Survival", ylab = "Actual Survival",main = "Calibration Curve")

**For predictions of the vilidation cohort**

library(rms)

fit1 <-glm(LB~LVSI+Ca12501+myometrialinvasion+histotypes+ER+Ki67+P5301,data = Ltraindata02,x=T,y=T)

pre<-predict(fit1,newdata = testdata)

predictions<-predict(fit1,newdata = testdata)

predictions

**For External Validation of Nomogram**

f2<-lrm(Ltestdata$LB~predictions,x=T,y=T)

validate(f2,method = "boot",B=1000,dxy=T)

**For Calibration Curve for Validation Cohort**

cal <- calibrate(f2,method = "boot",B = 1000)

plot(cal, xlab = "Nomogram Predicted Survival", ylab = "Actual Survival",main = "Calibration Curve")

**For Computing the C-Index and 95% CI in training cohort and vilidation cohort**

library(rms)

fit1 <- glm(LB~LVSI+Ca12501+myometrialinvasion+histotypes+ER+Ki67+P5301,data = Ltraindata02,family = "binomial")

pre <- predict(fit1,type='response')

plot.roc(Ltraindata02$LB, pre,

main="ROC Curve", percent=TRUE,

print.auc=TRUE,

ci=TRUE, of="thresholds",

thresholds="best",

print.thres="best")

rocplot1 <- roc(Ltraindata02$LB,pre)

ci.auc(rocplot1)

/////////////////////////////////////////

pre1 <- predict(fit1,newdata = Ltestdata02)

> plot.roc(Ltestdata02$LB, pre1,

main="ROC Curve", percent=TRUE,

print.auc=TRUE,

ci=TRUE, of="thresholds",

thresholds="best",

print.thres="best")

rocplot2 <- roc(Ltestdata02$LB,pre1)

ci.auc(rocplot2)
